# Toward surface defect detection in electronics manufacturing by an accurate and lightweight YOLO-style object detector

**DOI:** 10.1038/s41598-023-33804-w

**Published:** 2023-05-01

**Authors:** Jyunrong Wang, Huafeng Dai, Taogen Chen, Hao Liu, Xuegang Zhang, Quan Zhong, Rongsheng Lu

**Affiliations:** 1 Hefei University of Technology, Anhui, Hefei China; 2 LCFC (Hefei) Electronics Technology Co., Ltd., Anhui, Hefei China; 3 Hefei LCFC Information Technology Co., Ltd., Anhui, Hefei China; 4 Tsinghua University, Beijing, China

**Keywords:** Computational science, Computer science

## Abstract

In electronics manufacturing, surface defect detection is very important for product quality control, and defective products can cause severe customer complaints. At the same time, in the manufacturing process, the cycle time of each product is usually very short. Furthermore, high-resolution input images from high-resolution industrial cameras are necessary to meet the requirements for high quality control standards. Hence, how to design an accurate object detector with real-time inference speed that can accept high-resolution input is an important task. In this work, an accurate YOLO-style object detector was designed, ATT-YOLO, which uses only one self-attention module, many-scale feature extraction and integration in the backbone and feature pyramid, and an improved auto-anchor design to address this problem. There are few datasets for surface detection in electronics manufacturing. Hence, we curated a dataset consisting of 14,478 laptop surface defects, on which ATT-YOLO achieved 92.8% mAP0.5 for the binary-class object detection task. We also further verified our design on the COCO benchmark dataset. Considering both computation costs and the performance of object detectors, ATT-YOLO outperforms several state-of-the-art and lightweight object detectors on the COCO dataset. It achieves a 44.9% mAP score and 21.8 GFLOPs, which is better than the compared models including YOLOv8-small (44.9%, 28.6G), YOLOv7-tiny-SiLU (38.7%, 13.8G), YOLOv6-small (43.1%, 44.2G), pp-YOLOE-small (42.7%, 17.4G), YOLOX-small (39.6%, 26.8G), and YOLOv5-small (36.7%, 17.2G). We hope that this work can serve as a useful reference for the utilization of attention-based networks in real-world situations.

## Introduction

The problem of surface defect detection problem is important for quality control in electronics manufacturing. This problem has several characteristics. First, a real-time inference speed is required. During the manufacturing process, the cycle time of each product is usually very short. Second, the training time should be short. In real-world situations, the lifetimes of numerous products are very short. Furthermore, small and medium-sized enterprises typically do not have abundant computational resources. Third, high-resolution input is necessary. Due to the high-quality control standards required for electronic products, the samples used for quality control are collected by high-resolution industrial cameras. Although medium, large, or extra-large model architectures can achieve accurate performance, their long training times and slow inference times are prohibitive in real situations. Fourth, the designed model should be easy to deploy. Complicated operators are not directly supported by inference architectures. Hence, to address this problem, there is a critical need to design an accurate object detector with a real-time inference speed for high-resolution input images, a minimal training time, and a simple design.

Currently, You Only Look Once (YOLO)-style object detectors^1–3^ and fully convolutional one-stage (FCOS)-style object detectors^4,5^ are usually used for real-time object detection tasks. YOLO series and their variants have been widely used to various applications. Chen et al. proposed an improved YOLOv5 for plant disease recognition^6^. Dewi et al. proposed an improved YOLOv3 for small traffic sign recognition^7^. Dewi et al. proposed an improved YOLOv5 for road marking sign identification^8^. Mekhalf et al. conducted a comparison among YOLOv5, transformer, and EfficientDet on the task of crop circle detection in desert^9^. Zhang et al. proposed an improved YOLOv5 for target detection of forward-looking sonar image^10^. Xu et al. proposed an improved YOLOv5 for safety helmet wearing detection^11^. Yao et al. proposed an improved YOLOv5 for kiwifruit defects detection^12^. Dewi et al. proposed an improved YOLOv4 for advanced traffic sign recognition^13^. Among these elegant studies, the key issue is to design a fast and accurate object detector. To achieve fast and accurate object detection, (1) a good combination of local features and global features, (2) a more effective feature integration method^14^, (3) features which extracts from the foreground may have better performance than that extract from the whole image, and (4) a better initial solution are all beneficial. The latest YOLO-style object detector, YOLOv8^15^, is focused on a new backbone network, novel activation function, decoupled head, and anchor-free design to achieve fast and accurate object detection. Recently, self-attention-based networks have shown a superior ability to extract global features from input^16–18^. The self-attention module is designed such that the receptive field for the features is learnable instead of fixed^16–18^. Furthermore, the self-attention module can extract the features focusing on the foreground. However, such a self-attention module has more parameters than a convolutional module. An elegant proposal, TPH-YOLOv5^19^, uses several self-attention modules corresponding to multiple scales in a feature pyramid. TPH-YOLOv5^19^ performs well for object detection tasks focusing on remote sensing images. TPH-YOLOv5 use several self-attention modules to localize the objects from the high-density scenes. However, the large computational cost of TPH-YOLOv5^19^ makes it unsuitable use in surface defect detection in electronics manufacturing. On the other hand, the various scales of the objects of interest may necessitate feature extraction at more scales and feature integration by means of a feature pyramid^20^. Another elegant proposal, Scaled-YOLOv4^20^, has been the subject of some experiments focusing on this consideration. However, for object detectors with such a many-scale design, the default auto-anchor method may not perform well. Hence, an improved auto-anchor search to enhance the initial solution is important to alleviate this problem with many-scale designs, which can cause poor performance in terms of the best possible recall^4,5^ and result in a poor initial solution for object detection tasks.

In this work, we propose an accurate object detector, ATT-YOLO (attention-YOLO), that is oriented toward the problem of surface defect detection in electronics manufacturing. ATT-YOLO uses only one self-attention module^21^ to achieve better global feature extraction and localize the interested objects from the highly density scenes^10^ at the cost of fewer additional parameters compared with methods that use multiple self-attention modules. ATT-YOLO also uses a many-scale backbone and feature pyramid as well as an improved auto-anchor design to obtain better initial solutions. For the problem under consideration, we used a small-scale YOLOv5 model as our baseline model to conduct a series of experiments. We verified our design on both a self-curated dataset and the COCO benchmark dataset^22^.

## Results

### Ablation study of ATT-YOLO

We designed a series of experiments to analyze the contribution of each aspect of the design. The results are shown in Table 1.

**Table 1:** Ablation study of ATT-YOLO on the COCO dataset.

Exp	Description	Epochs	mAP0.5:0.95 (%)	Training time (h)
A	YOLOv5-small + Mish	300	26.8	247.0
B	A + deeper multiscale backbone + deeper feature pyramid + self-attention module	300	33.9	329.5
C	B + improved auto-anchor design	300	34.9	329.5
D	The same settings as experiment C except that the Mish activation function is replaced by the SiLU activation function	300	35.3	296.0
E	The same model as in experiment C but with 400 epochs	400	35.3	424.0
F*	A + deeper multiscale backbone + deeper feature pyramid except that the Mish activation function is replaced by the SiLU activation function	300	43.3	–
G	The same settings as experiment D but with 400 epochs and training from scratch	400	44.9	395.0

*Means the data cited from the YOLOv5. ‘–’, means the data not available.

The interpretation of these results can be divided into four salient points. (1) Regarding the activation function, we compare Mish^23^ and SiLU^24^ in combination with both the baseline model (YOLOv5-small) and ATT-YOLO. The results show that for both model designs, the SiLU activation function can achieve a higher value of the mean average precision (mAP0.5:0.95) index with less training time. (2) In experiment B, the deeper multiscale backbone, deeper feature pyramid, and self-attention module can improve the mAP0.5:0.95 performance by 8.1% compared to that of the baseline model. (3) The results of the auto-anchor comparison (Exp. B and Exp. E) show that the improved auto-anchor design can increase the mAP0.5:0.95 by 1.4% compared to that of the model without the improved auto-anchor design. (4) Comparisons of the results obtained with different numbers of training epochs ((Exp. C, Exp. E) and (Exp. D, Exp. G)) show that when the self-attention module is included, training for more epochs is better than training for fewer epochs. In contrast, object detectors using only convolution operations usually converge within 300 epochs^14,25^. Comparisons between the with/without the self-attention module is shown in the (Exp. F, Exp. G). The self-attention module improves 1.6% in terms of the mAP0.5:0.95. Finally, the results obtained using the ATT-YOLO model design, the improved auto-anchor design, the SiLU activation function and more training epochs are the best (mAP0.5:0.95 44.9%) among all experiments in the ablation study.

The possible reason that why the default auto-anchor of YOLOv5 is not suitable for the ATT-YOLO may cause from the simplified genetic algorithm and the design of fitness function which caused the limited fitting ability of an optimization problem. The default YOLOv5 has only three prediction heads, but the ATT-YOLO has six prediction heads. The parameters needed to be optimized is more than original network design. Since the genetic algorithm has random strategy during the algorithm execution, it cannot be guaranteed the next generation results are better than previous results. Hence, without the elitism design in the genetic algorithm, the optimized results may worse than previous generation. Another possible reason of the fitness function design, the IOU distance is more directly to the object detection task than the Euclidean distance. Since the objective of the object detection task is the IOU metric or its variant, directly used the IOU distance as the fitness function of the designed genetic algorithm may have better optimized results in our experience. In the results of this work, the default auto-anchor method BPR is 11.4%, the improved auto-anchor method improves BPR from 11.4% to 99.9% and improved mAP0.5:0.95 from 33.9% to 34.9%. The experiment results are consistent with our experience.

### Comparison with existing lightweight and state-of-the-art YOLO-style object detectors

**Figure 1:**
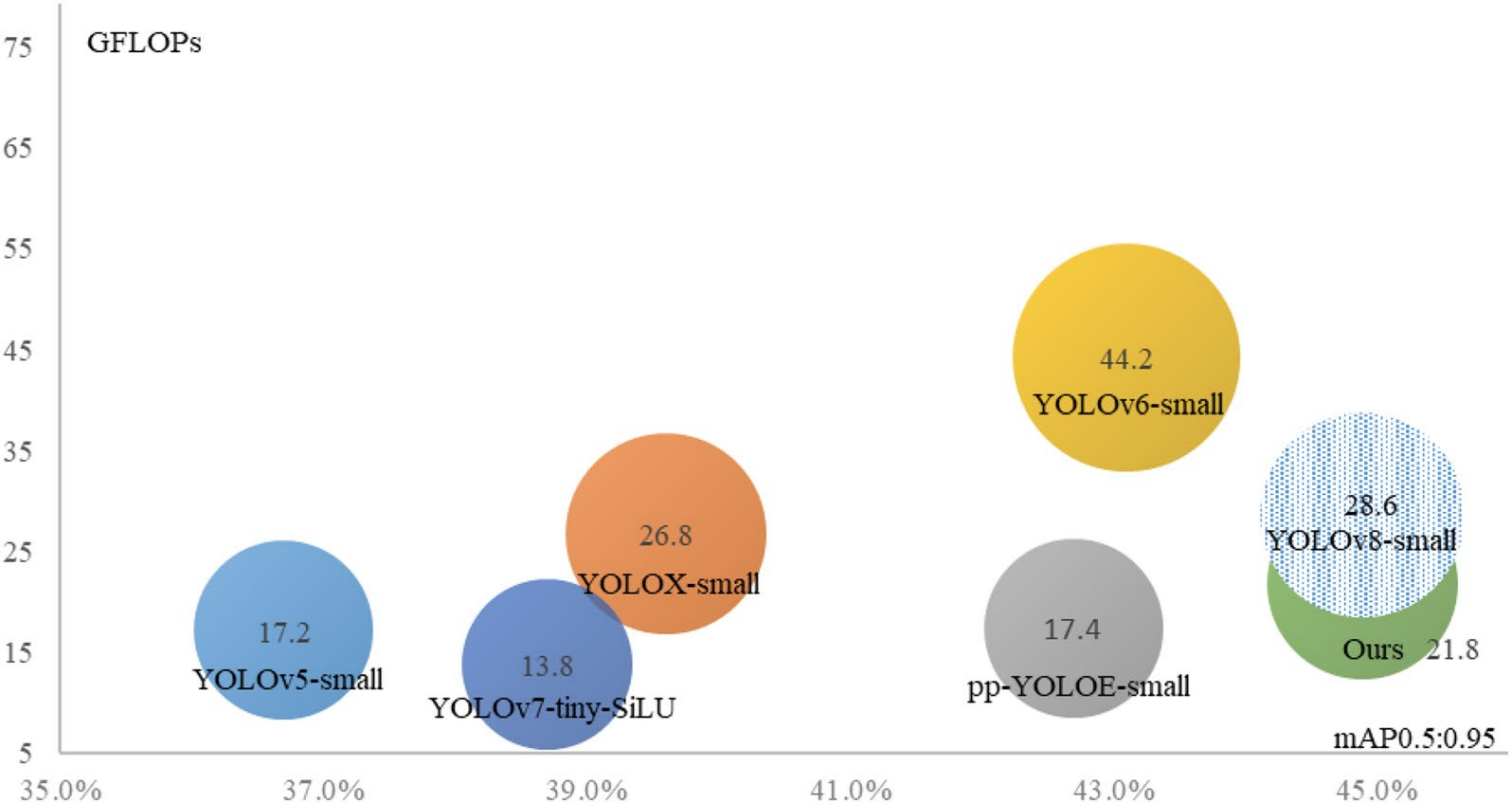
Comparison among existing YOLO-style object detectors in terms of mAP0.5:0.95 on the COCO dataset and computational cost (GFLOPs). A smaller number of GFLOPs and a larger mAP0.5:0.95 are better. The x-axis represents mAP0.5:0.95, and the y-axis represents the computational cost (GFLOPs). The area of the circle representing each model is also proportional to its computational cost. This figure was plotted by Microsoft Office 2016.

In this section, we compare ATT-YOLO with other methods with similar computational costs, namely, small-scale YOLO designs based on existing YOLO-style object detectors. The compared lightweight and state-of-the-art models include YOLOv5-small^26^, YOLOX-small^27^, pp-YOLOE-small^28^, YOLOv6-small^25^, YOLOv7-tiny-SiLU^14^, and YOLOv8-small^15^ (Fig. 1 and Table 2).The results show that ATT-YOLO achieves a mAP0.5:0.95 of 44.9% and GFLOPs of 21.8G, outperforming some state-of-the-art YOLO-style object detectors with similar computational costs. The related experimental results are cited from the corresponding papers. The detailed results are shown in Table 2 and Fig. 1.

**Table 2:** A comprehensive comparison of small-scale and state-of-the-art YOLO-style models on the COCO dataset.

Model	FLOPs	mAP0.5:0.95 (%)	#Param (M)	mAP0.5 (%)	AP_s_ (%)	AP_m_ (%)	AP_l_ (%)
YOLOv5-small^26^	17.2G	36.7	7.3	55.4	–	–	–
YOLOX-small^27^	26.8G	39.6	9.0	–	–	–	–
pp-YOLOE-small^28^	17.4G	42.7	7.9	60.5	23.2	46.4	56.9
YOLOv6-small^25^	44.2G	43.1	17.2	–	–	–	–
YOLOv7-tiny-SiLU^14^	13.8G	38.7	6.2	56.7	18.8	42.4	51.9
YOLOv8-small^15^	28.6G	44.9	11.2	–	–	–	–
Ours	21.8G	44.9	23.1	63.2	29.7	49.6	57.6

‘-’, Means the corresponding performance are not available on the corresponding proposals.

### Results on the LCFC-Laptop dataset

In this section, to verify the ability to handle the electronics manufacturing surface detection problem, we present two experiments conducted on the LCFC-Laptop dataset: one focuses on the multiclass object detection task, and the other focuses on the binary-class object detection task. The multiclass object detection task can be used to analyze which defect class is predominant during a certain period. Since each defect has corresponding reasons for its occurrence, the analysis of multiclass object detection can be employed to improve the manufacturing process. On the other hand, the binary-class object detection task focuses on determining whether a given sample is defective or not, which is the commonly used industry standard for a production line. It provides overall metrics for product quality. By combining all classes of defects into the "defect" class, the imbalanced problem in the LCFC-Laptop dataset can be alleviated.

**Figure 2:**
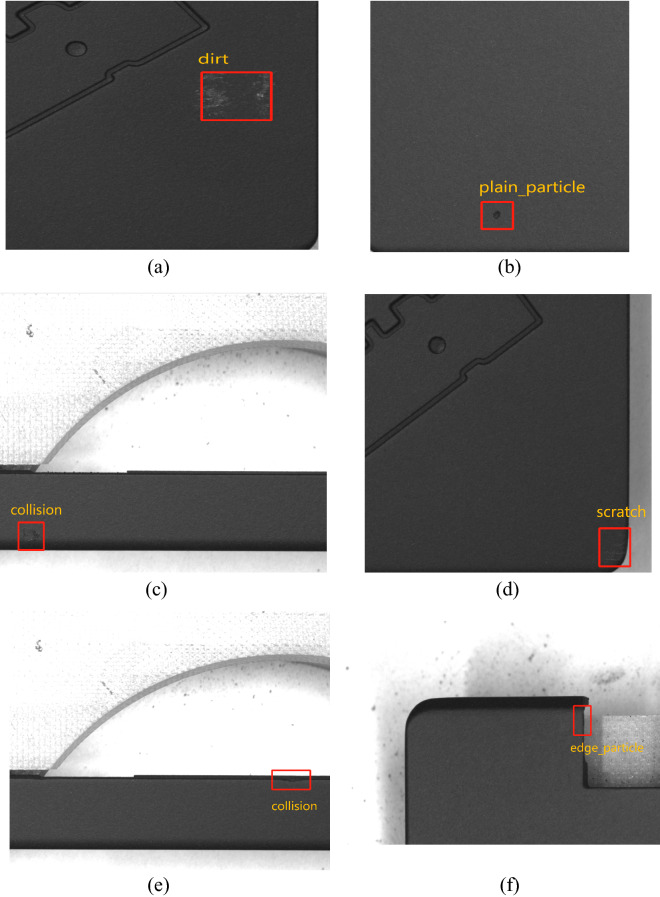
The prediction results of ATT-YOLO on various defect types. (**a**) Dirt, (**b**) plain particle, (**c**) collision, (**d**) scratch, (**e**) collision, and (**f**) edge particle. This figure was plotted by python with version 3.8.12.

In the multiclass task (Table 3), ATT-YOLO achieves an overall precision of 84.7%, an overall recall of 87.4%, and a mAP0.5 of 90.3%. On the other hand, ATT-YOLO achieves an overall precision of 88.2%, an overall recall of 88.1%, and a mAP0.5 of 92.8% on the binary-class object detection task (Table 4). Some prediction results can be seen in the Fig. 2. These results show that ATT-YOLO may overcome the data imbalanced problem to some degree. However, ATT-YOLO achieves a higher mAP0.5 value on the binary-class object detection task than on the multiclass object detection task. As shown in Table 5, on the single RTX 3080 GPU, ATT-YOLO can achieve an inference speed of 111.0 FPS based on the TensorRT architecture, whereas the inference speed of the PyTorch implementation can reach 65.3 FPS on the single TITAN RTX GPU. Both inference speeds are sufficient to meet the standard for real-time prediction on regular GPUs instead of high-end GPUs. However, some mislabeling problems in this dataset may have detrimental impacts on an object detector. Despite the noisy labels in the dataset, ATT-YOLO can achieve good results on this dataset. However, to meet the high standards of quality control, it will be necessary to evolutionarily refine this dataset. As mentioned above, the binary class object detection task alleviates the imbalanced problem in the LCFC-Laptop dataset, which improves the overall mAP0.5 from 90.3% to 92.8%. However, the multiclass object detection task is beneficial for analyzing the majority class(es). After changing the manufacturing process, the quantity of a given change to improve the production line's yield rate can be estimated. The feedback between changes to the manufacturing process and the yield rate is valuable for optimizing the manufacturing process. The main training parameters are listed in Table 6. Both the multiclass object detection task and the binary class object detection task require 8 h of training time. The primary training environment used for the LCFC-Laptop dataset includes PyTorch 1.7.0, opencv-python 4.1.1, numpy 1.18.5, and Pillow 7.1.2. The detailed list of Python packages can be found on our page.

**Table 3:** Performance in the multiclass object detection task on the LCFC-Laptop dataset.

Defect type	Precision (%)	Recall (%)	mAP0.5 (%)
Dirt	87.4	81.7	89.3
Plain particle	83.2	94.3	93.7
Edge particle	79.2	85.7	87.0
Collision	80.7	72.4	78.6
Scratch	89.8	94.7	96.2
Unknown	88.2	95.8	96.9
All	84.7	87.4	90.3

**Table 4:** Performance in the binary class object detection task on the LCFC-Laptop dataset.

Defect type	Precision (%)	Recall (%)	mAP0.5 (%)
All	88.2	88.1	92.8

**Table 5:** Inference speed of ATT-YOLO on the LCFC-Laptop dataset.

Architecture	FPS	GPU
PyTorch	65.3	The TITAN RTX GPU
TensorRT	111.0	The RTX 3080 GPU

**Table 6:** ATT-YOLO training parameters.

Parameter	Value	Parameter	Value
lr0	0.0032	lrf	0.12
Momentum	0.843	weight_decay	0.00036
Mosaic	1.000	mixup	0.243

## Discussion

In this study, to demonstrate the ability to handle the electronics manufacturing surface defect detection problem, we conducted a series of experiments to verify each component of the ATT-YOLO design. Because of the need for simplicity in this problem, we used only a simple design for the model architecture. We believe that by replacing the simple convolutional and attention modules used in ATT-YOLO with more sophisticated, novel designs, it may be possible to achieve better performance than that reported here. To save computational costs, we did not perform hyperparameter tuning in this study, and we trained our models from scratch without using another external dataset. We further compared ATT-YOLO against other YOLO-style object detectors with similar computational costs on the COCO dataset. The results show that ATT-YOLO outperforms existing YOLO-style object detectors under the condition of a similar computational cost. Furthermore, to evaluate the ability to handle the problem of interest, we conducted two experiments on a real-world dataset. Despite the noisy labels in the dataset, ATT-YOLO achieved good results in these experiments. These results show that ATT-YOLO may be suitable for handling this real-world problem.

In the future, first, it will be necessary to evolutionarily refine the LCFC-Laptop dataset for benchmarking performance on the surface defect detection problem in electronics manufacturing. Second, a more efficient and accurate design of ATT-YOLO for this problem should be pursued. Third, unsupervised or semisupervised methods should be developed to reduce the cost of dataset collection. We believe that the availability of a sufficiently high-quality dataset will help improve performance to meet the high standards required in the industry.

## Materials and methods

### The LCFC-laptop dataset

LCFC (Hefei) Electronics Technology Co., Ltd. (LCFC) is a wholly owned subsidiary of Lenovo. Worldwide, one out of eight laptops sold are manufactured by LCFC; the cumulative number of laptops sold has reached 0.2 billion, and over 126 countries have bought laptops from Lenovo. Hence, through collaboration with this company, we were able to gain the opportunity to establish an extensive laptop dataset to verify the performance of ATT-YOLO.

**Figure 3:**
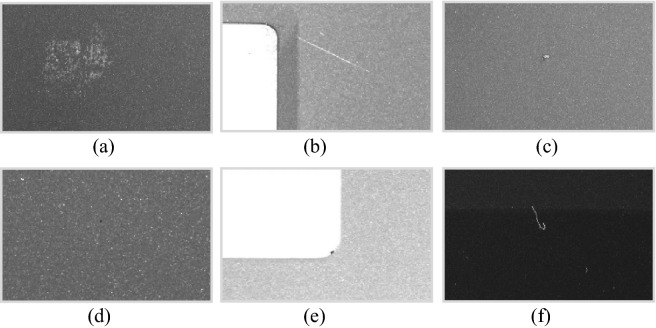
Examples of each defect type. The defect types include (**a**) dirt, (**b**) scratches, (**c**) collision defects, (**d**) plain particles, (**e**) edge particles, and (**f**) defects of unknown type. This figure presents only the regions of interest of the original input images. This figure was plotted by python with version 3.8.12.

To construct the LCFC-Laptop dataset, we collected samples acquired by four 5000 × 5000 high-resolution industrial cameras with 6 sets of lights, including white lights, blue lights, and red lights. We believe that a given defect is associated with a combination of specific wavelengths and that illumination with a broad spectrum can allow a variety of defects to be well captured. Hence, we used various wavelengths of light to collect this dataset. The 6 sets of lights were tuned by senior optical engineers. Next, we used the same standards adopted by the senior engineers and quality inspectors of LCFC to label this dataset. Finally, we obtained a dataset consisting of 14,478 annotated defects. The detailed statistics of this dataset are shown in Table 7. The dataset includes various types of defects, such as scratches, dirt, plain particles, edge particles, collision defects, and defects of unknown type. Examples of each defect type are shown in Fig. 3. Although the dataset was labeled by senior engineers and quality inspectors, some mislabeled samples are inevitable. The mislabeled samples can be divided into three categories. First, a sample of one class may be labeled as belonging to another class. Second, a nondefect sample may be labeled as a defect sample. Third, a defect sample may be labeled as a nondefect sample. Since the samples in this dataset will be on the market within 6–12 months, this dataset analyzed during the current study available from the corresponding author on reasonable request. After all these products are on the market, the download link of this dataset will be available at the URL.

**Table 7:** Statistics of the LCFC-Laptop dataset.

Defect type	Number of defects
Dirt	11,285
Plain particle	605
Edge particle	35
Collision	29
Scratch	1104
Unknown	1420
All	14,478

### Data preprocess of the LCFC-Laptop dataset

**Figure 4:**
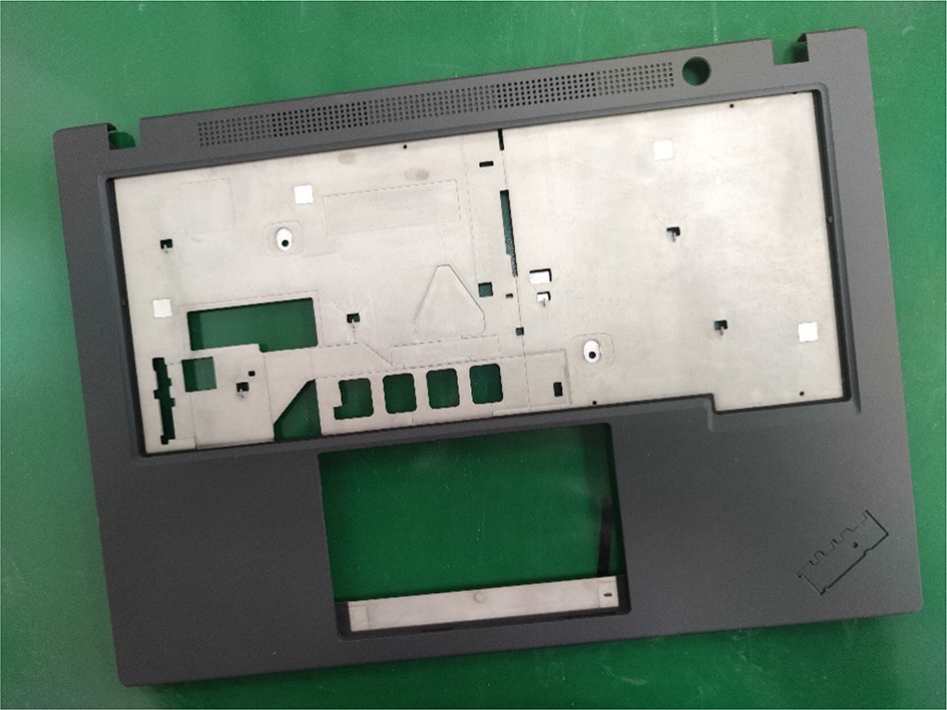
The appearance of a portable computer shell. The black regions represent regions of interest. The corresponding parts for the monitor and touchpad are considered background in the context of surface defect detection on portable computer shells. This figure was plotted by the Dahua-A5B57MG200 camera.

**Figure 5:**
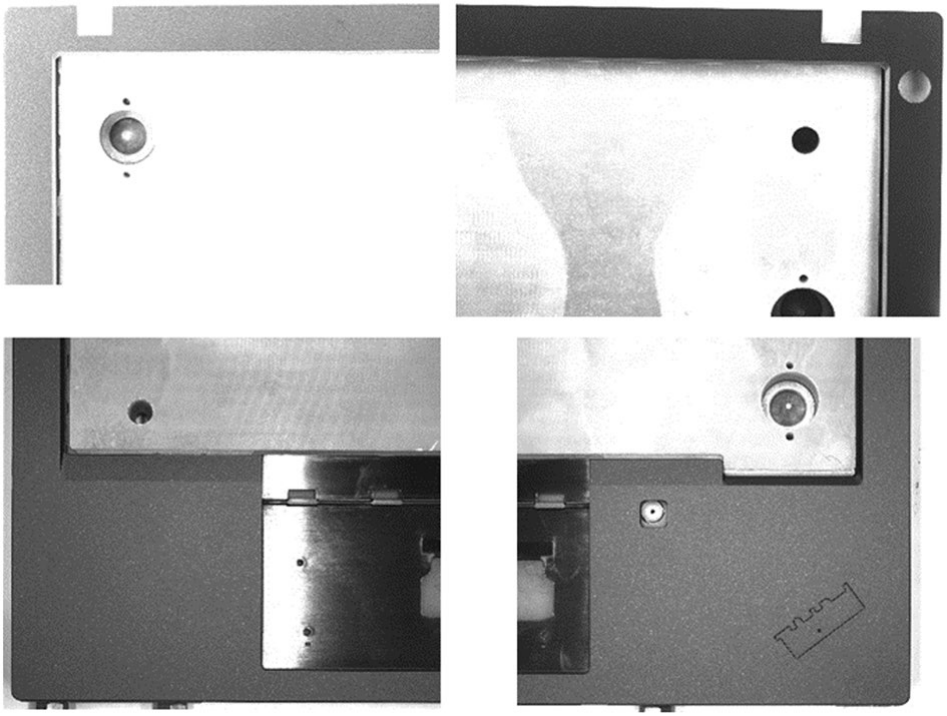
The original sample images have a resolution of 5000 × 5000, captured by an industrial camera. To obtain high-quality images from these high-resolution industrial images, we use four high-resolution cameras to capture the top left, top right, bottom left, and bottom right sections of an original sample. This figure was plotted by the python with version 3.8.12.

**Figure 6:**
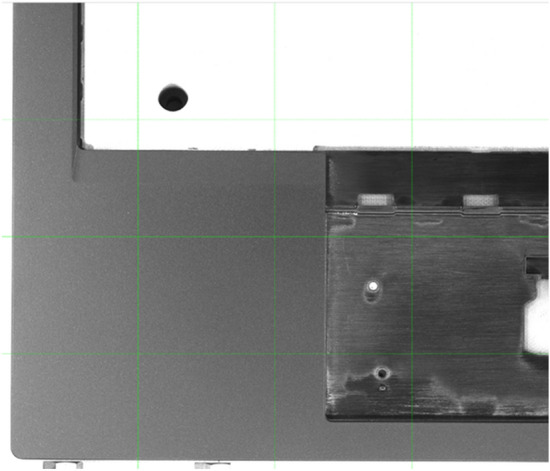
Illustration of dividing the original images into 1280 × 1280 sub-images. We split the original images into 1280 × 1280 sub-images along the green line. The two adjacent sub-images have an overlapping area of 32 pixels to prevent defects from being damaged during the process. This figure was plotted by the ImageLabeler (https://github.com/Manchery/ImageLabeler).

The LCFC-Laptop dataset consists of images collected from portable computer shells (Fig. 4). We use four high-resolution cameras to capture photos of a portable computer shell with a resolution of 5000 × 5000 pixels (Fig. 5). Due to the high GPU memory requirements for training a model with such high-resolution images, we divide the original image into smaller pieces. As shown in Fig. 6, the original image is split into 16 smaller sub-images. To prevent defects from being damaged during the process, there are 32-pixel overlapping regions at the edges of two adjacent sub-images. Since defects smaller than 32 pixels are difficult to distinguish with the naked eye, the LCFC-Laptop dataset ultimately consists of 1280 × 1280 images with 32-pixel overlapping regions.

### The COCO dataset

The Common Objects in Context (COCO) dataset^22^ is one of the most popular large-scale labeled image datasets available for public use. It contains various types of objects that are encountered on a daily basis and image annotations for 80 object classes, with over 1.5 million object instances. The COCO dataset is used for multiple computer vision tasks, such as object detection, instance segmentation, and image captioning. Its versatility and multipurpose scene variations make it highly suitable for training a computer vision model and benchmarking its performance.

### A brief review of the baseline object detector

YOLO is one of the most widely known object detection algorithms due to its speed and accuracy^14,25^. YOLOv5 implements a family of object detection architectures and provides several pretrained models that have been trained on the COCO dataset^26^. YOLOv5 is designed with a flexible architecture that allows developers and researchers to modify and extend it with customized layers and model architectures. Furthermore, YOLOv5 and its community provide a series of sample codes for various deployment architectures, such as ONNXRuntime, TensorRT^29^, and TensorFlow. To address the problem under consideration, we chose the YOLOv5-small model with version 5.0 as our baseline model. 

YOLOv5 consists of three parts which are the backbone module, the neck module, and the prediction head module. On the backbone design of YOLOv5, the modified CSPDarknet^30,31^ are used, which by combination use of the Focus module, the BottleneckCSP module^30,31^, and the SPP module (Spatial Pyramid Pooling)^32^. On the neck module design, PANet (Path Aggregation Network) are used for information fusion. On the prediction head module, classification and regression are coupled which same as the YOLOv3 design^3^ of the prediction head module. 

### Design of ATT-YOLO

**Figure 7:**
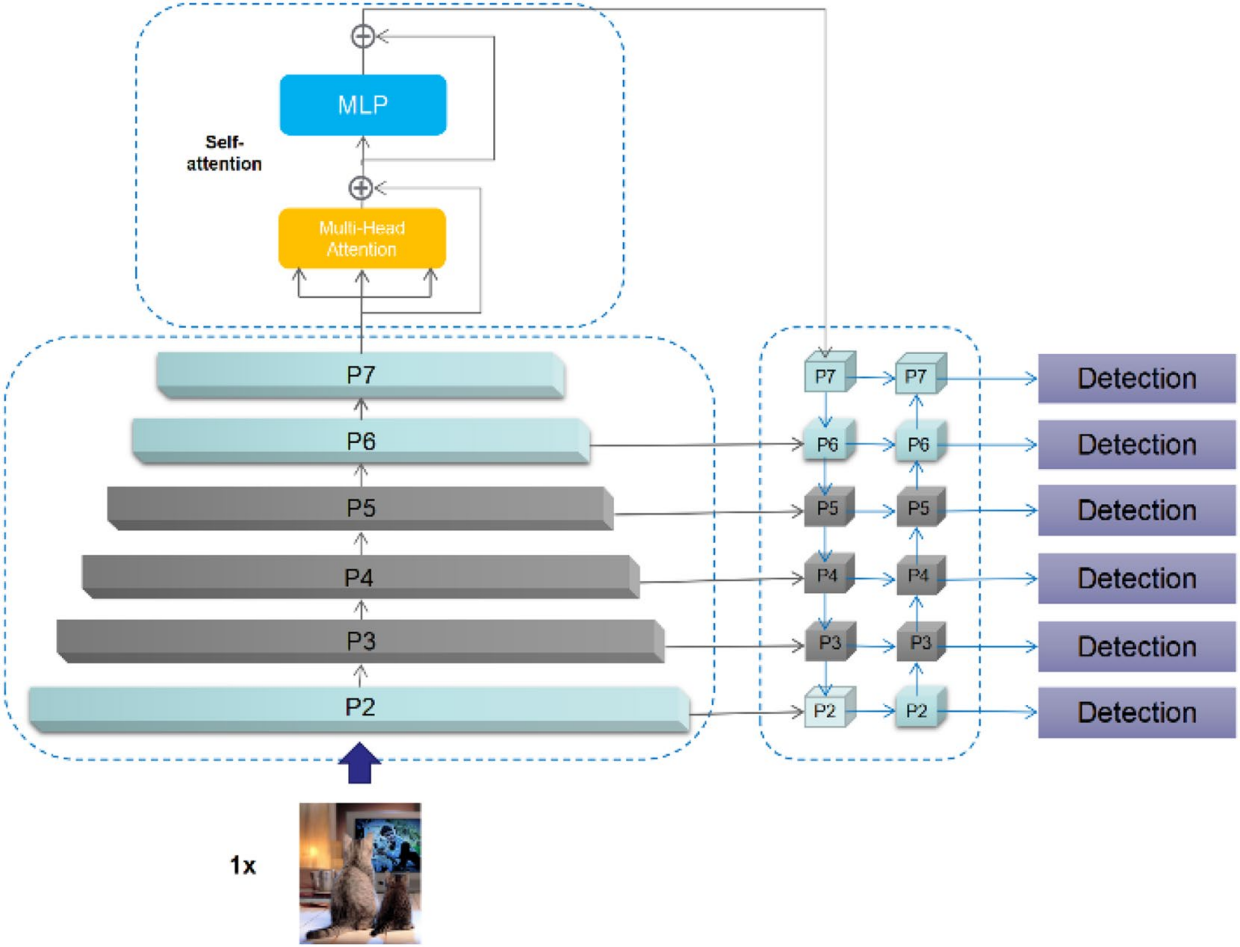
The design of ATT-YOLO. ATT-YOLO uses a many-scale backbone and feature pyramid. In the backbone, ATT-YOLO uses only one self-attention module, which is located immediately behind the P7 module. The gray blocks represent elements from the original design of YOLOv5. This figure was plotted by the Microsoft Office2016.

According to our observations, (1) a surface defect detection dataset usually contains objects with complicated shapes and at many scales, (2) few YOLO-style object detectors use global features to enhance the performance of object detection^14,25^, and (3) a many-scale design may cause the default auto-anchor method to not perform well. Hence, the design of ATT-YOLO consists of three parts. First, ATT-YOLO uses a many-scale backbone and a many-scale feature pyramid. Second, ATT-YOLO includes only one self-attention module, which is located behind the seventh convolutional module in the backbone. Third, ATT-YOLO uses an improved auto-anchor method to obtain a set of better initial solutions. To meet the requirements of the problem at interest, only a small-scale model was used in this work to conduct the related experiments, and only simple convolutional and self-attention modules were adopted for easy deployment. Nevertheless, despite the use of small-scale models, the training time spent on these models ranged from 247.0 to 424.0 h. In these experiments, training was conducted mainly on eight RTX 3090 GPUs in a distributed manner. All hyperparameters used in ATT-YOLO were the same as those in the default YOLOv5 model. The detailed design of ATT-YOLO is shown in Fig. 7. The backbone of ATT-YOLO consists of seven convolutional modules and one self-attention module, which is immediately behind the last convolutional module (P7). The feature pyramid design of ATT-YOLO spans from the P2 module to the P7 module and is adapted from the design of PANet^33^. The detection head used in this work is the original design used in YOLOv5^26^.

The actual backbone design implementation between ATT-YOLO and YOLOv5-small is shown in the Table 8, the basic module is same to the YOLOv5-small consisting of Conv + C3, another difference between the two models is the final module, ATT-YOLO adds self-attention behind the P7 module instead of the original implementation (Conv + SPP + C3). The self-attention module is implemented by the multi-head attention and MLP. The connection of backbone to the feature pyramid and the detection head is shown in the Fig. 7.

**Table 8:** The comparison between backbones of ATT-YOLO and YOLOv5-small.

Module	ATT-YOLO	YOLOv5-small
P2	Focus	Focus
P3	Conv + C3	Conv + C3
P4	Conv + C3	Conv + C3
P5	Conv + C3	Conv + SPP + C3
P6	Conv + C3	–
P7	Conv + SPP + C3 + self-attention	–

‘–’, Means the default YOLOv5-small model without the P6 and P7 modules.

As a result, ATT-YOLO has a computational cost of 21.8 GFLOPs, which is slightly higher than the original computational cost of the YOLOv5-small model (17.2 GFLOPs). Since the CSPDarknet architecture^30,31^ was proposed by the YOLOv4 authors^34^, the original activation function used in this architecture is Mish^23^. On the other hand, the original activation function used in the YOLOv5 design^26^ is the sigmoid linear unit (SiLU) function^24^. Hence, this work compares these two activation functions in both the original YOLOv5-small model and the ATT-YOLO design. The detailed experimental design, including the activation function comparisons, is summarized in Table 1. 

To reveal the efficiency of the ATT-YOLO design and to reduce the difficulty of deployment in real situations, this work relies on simple convolutional modules, a simple self-attention module^21^, and the original settings of the small-scale YOLOv5-small model. We believe that other types of convolutional modules, such as fast Fourier convolution^35,36^ or dilated convolution^37^, may offer better performance than these simple convolutional modules. Similarly, regarding attention module selection, recent variants of Transformers such as Swin Transformer^16,17^, DETR^38^, Deformable DETR^39^, DINO^40^, and ViT-Adapter-B^41^ may offer better performance than our simple self-attention module^21^. Furthermore, to save computational costs, we did not perform hyperparameter tuning in this work. Hence, it may be possible to improve the results of this work by performing a suitable hyperparameter search. To address the need for high-resolution input images that is characteristic of the problem of interest, ATT-YOLO was implemented with an input size of 1280 × 1280 for these experiments. The 1280 × 1280 input images were sliced from the original 5000 × 5000 input images, with some overlap between slices to avoid some defects being cut into different parts.

### An improved auto-anchor method

According to our abundant experience from the intelligent computing lab on the evolutionary algorithms and the genetic algorithms and applications on several fields^42–48^. We improve the default YOLOv5 auto-anchor design. The default YOLOv5 auto-anchor design uses a simple genetic algorithm in cooperation with the kmeans algorithm. The kmeans algorithm in YOLOv5 uses the Euclidean distance as a fitness function to evolve a set of initial anchors. This design can perform well with the default YOLOv5 backbone and feature pyramid, achieving a best possible recall (BPR)^4,5^ of 99.9% with the default model architecture. The BPR is defined as the ratio of the maximum number of ground-truth boxes a detector can recall to the total number of ground-truth boxes^4,5^. However, when the default auto-anchor design^26^ is directly applied in ATT-YOLO, the value of the BPR is reduced to 11.4%, which may imply a poor initial solution for the object detection task that used for handling this problem in electronics manufacturing. 

In this work, ATT-YOLO uses a simple genetic algorithm and an elitism mechanism to ensure that the offspring results are always better than the ancestor results. We also use a design in which a genetic algorithm works in cooperation with the kmeans algorithm. However, ATT-YOLO uses the intersection-over-union (IOU) distance instead of the Euclidean distance as the fitness function. The IOU distance is calculated as the 1-IOU. For simplicity, this work uses the original IOU definition instead of any novel IOUs^24,49–51^, which may offer better performance but require more complicated computations. As a result, the value of the BPR improves from 11.4 to 99.9% with the ATT-YOLO design.

## Conclusions

The object detection task can be applied to numerous scenarios, each with its own unique requirements. In this work, we have summarized the requirements for surface defect detection in electronics manufacturing, where both inference speed and accuracy of the object detector are essential. In this context, ATT-YOLO provides the best trade-off among existing YOLO-style object detectors. However, when the primary focus of a specific scenario is the accuracy of the object detector, YOLO-style object detectors with larger computational costs and transformer-based object detectors emerge as superior choices for that scenario.

In this work, ATT-YOLO uses many-scale backbone and feature pyramid, an improved auto-anchor method, and a self-attention module to design object detector for surface defect detection in electronics manufacturing. We verified ATT-YOLO by the self-curated LCFC-Laptop dataset and the COCO benchmark dataset. As a result, ATT-YOLO satisfies the reequipments of surface defect detection and achieve the best tradeoff among lightweight YOLO-style object detectors.

## Future work

Since some mislabeling issues are inevitable in human-curated datasets, it is essential to continually refine the LCFC-Laptop dataset. Additionally, collecting defect data from various materials is crucial for extending this work.

On the other hand, the supervised method proposed in this work targets scenarios where sufficient defect data is available. Due to the low probability of defects occurring, it can be challenging to collect adequate data to train a high-performing model using a supervised approach. Therefore, it is important to develop unsupervised or semisupervised methods for situations where defect data is insufficient. These methods rely on a vast amount of normal data to capture the information and distribution of what is considered "normal." Once these models are trained, they use deviations from the normal data to determine whether a given input is normal or not.

## Data Availability

Since the samples in this dataset will be on the market within 6–12 months, this dataset analyzed during the current study available from the corresponding author on reasonable request. After all these products are on the market, the download link of this dataset will be available at the URL which can be available at https://bitbucket.org/att-yolov5/att-yolov5/src/main/.

## References

[1] Redmon, J., Divvala, S., Girshick, R. & Farhadi, A. in *Proceedings of the IEEE conference on computer vision and pattern recognition.* 779–788.

[2] Redmon, J. & Farhadi, A. in *IEEE Conference on Computer Vision & Pattern Recognition.* 6517–6525.

[3] Redmon, J. & Farhadi, A. J. a. e.-p. YOLOv3: An Incremental Improvement. (2018).

[4] Tian, Z., Shen, C., Chen, H., He, T. J. I. T. o. P. A. & Intelligence, M. FCOS: A simple and strong anchor-free object detector. 1–1 (2020). 10.1109/TPAMI.2020.3032166 33074804

[5] Tian, Z., Shen, C., Chen, H. & He, T. in *2019 IEEE/CVF International Conference on Computer Vision (ICCV).*

[6] Chen Z (2022). Plant disease recognition model based on improved YOLOv5. Agronomy.

[7] Dewi, C., Chen, R.-C., Yu, H. & Jiang, X. Robust detection method for improving small traffic sign recognition based on spatial pyramid pooling. *J. Ambient Intell. Hum. Comput.* 1–18 (2021).

[8] Dewi C, Chen R-C, Zhuang Y-C, Christanto HJ (2022). Yolov5 series algorithm for road marking sign identification. Big Data Cogn. Comput..

[9] Mekhalfi ML (2021). Contrasting YOLOv5, transformer, and EfficientDet detectors for crop circle detection in desert. IEEE Geosci. Remote Sens. Lett..

[10] Zhang H, Tian M, Shao G, Cheng J, Liu J (2022). Target detection of forward-looking sonar image based on improved yolov5. IEEE Access.

[11] Xu, Z., Zhang, Y., Cheng, J. & Ge, G. in *Journal of Physics: Conference Series.* 012038 (IOP Publishing).

[12] Yao J (2021). A real-time detection algorithm for Kiwifruit defects based on YOLOv5. Electronics.

[13] Dewi C, Chen R-C, Liu Y-T, Jiang X, Hartomo KD (2021). Yolo V4 for advanced traffic sign recognition with synthetic training data generated by various GAN. IEEE Access.

[14] Wang, C.-Y., Bochkovskiy, A. & Liao, H.-Y. M. YOLOv7: Trainable bag-of-freebies sets new state-of-the-art for real-time object detectors. Preprint https://arxiv.org/abs/2207.02696 (2022).

[15] Glenn jocher *et al*. *YOLOv8. *https://github.com/ultralytics/ultralytics (2023).

[16] Liu, Z. *et al.* Swin Transformer: Hierarchical Vision Transformer using Shifted Windows. (2021).

[17] Liu, Z. *et al.* Swin Transformer V2: Scaling Up Capacity and Resolution. (2021).

[18] Roh, B., Shin, J. W., Shin, W. & Kim, S. J. a. e.-p. Sparse DETR: Efficient End-to-End Object Detection with Learnable Sparsity. (2021).

[19] Zhu, X., Lyu, S., Wang, X. & Zhao, Q. in *Proceedings of the IEEE/CVF International Conference on Computer Vision.* 2778–2788.

[20] Wang, C. Y., Bochkovskiy, A. & Liao, H. in *Computer Vision and Pattern Recognition.*

[21] Dosovitskiy, A. *et al.* An image is worth 16x16 words: Transformers for image recognition at scale. Preprint https://arxiv.org/abs/2010.11929 (2020).

[22] Lin, T. Y., Maire, M., Belongie, S., Hays, J. & Zitnick, C. L. J. S. I. P. Microsoft COCO: Common Objects in Context. (2014).

[23] Misra, D. Mish: A self regularized non-monotonic neural activation function. Preprint https://arxiv.org/abs/1908.08681 **4**, 10.48550 (2019).

[24] Du, S., Zhang, B., Zhang, P. & Xiang, P. in *2021 IEEE 2nd International Conference on Pattern Recognition and Machine Learning (PRML).* 92–98 (IEEE).

[25] Li, C. *et al.* YOLOv6: a single-stage object detection framework for industrial applications. Preprint https://arxiv.org/abs/2209.02976 (2022).

[26] Glenn jocher *et al*. *YOLOv5: *https://github.com/ultralytics/yolov5 (2021).

[27] Ge, Z., Liu, S., Wang, F., Li, Z. & Sun, J. YOLOX: Exceeding YOLO Series in 2021. (2021).

[28] Xu, S. *et al.* PP-YOLOE: An evolved version of YOLO. (2022).

[29] al., X.-Y. W. e. *TensorRTx: *https://github.com/wang-xinyu/tensorrtx (2020).

[30] Wang, C. Y. *et al.* CSPNet: A New Backbone that can Enhance Learning Capability of CNN. (2019).

[31] Wang, C. Y., Liao, H., Wu, Y. H., Chen, P. Y. & Yeh, I. H. in *2020 IEEE/CVF Conference on Computer Vision and Pattern Recognition Workshops (CVPRW).*

[32] He K, Zhang X, Ren S, Sun J (2015). Spatial pyramid pooling in deep convolutional networks for visual recognition. IEEE Trans. Pattern Anal. Mach. Intell..

[33] Liu, S., Qi, L., Qin, H., Shi, J. & Jia, J. in *2018 IEEE/CVF Conference on Computer Vision and Pattern Recognition (CVPR).*

[34] Bochkovskiy, A., Wang, C. Y. & Liao, H. YOLOv4: Optimal Speed and Accuracy of Object Detection. (2020).

[35] Nair, V., Chatterjee, M., Tavakoli, N., Namin, A. S. & Snoeyink, C. Fast Fourier transformation for optimizing convolutional neural networks in object recognition. Preprint https://arxiv.org/abs/2010.04257 (2020).

[36] Riaz, H. U. M., Benbarka, N. & Zell, A. in *2020 25th International Conference on Pattern Recognition (ICPR).* 7833–7840 (IEEE).

[37] Wei, Y. *et al.* in *Proceedings of the IEEE conference on computer vision and pattern recognition.* 7268–7277.

[38] Carion, N. *et al.* in *European conference on computer vision.* 213–229 (Springer).

[39] Zhu, X. *et al.* in *International Conference on Learning Representations.*

[40] Zhang, H. *et al.* DINO: DETR with Improved DeNoising Anchor Boxes for End-to-End Object Detection. (2022).

[41] Chen, Z. *et al.* Vision Transformer Adapter for Dense Predictions. (2022).

[42] Ho S-Y, Shu L-S, Chen J-H (2004). Intelligent evolutionary algorithms for large parameter optimization problems. IEEE Trans. Evol. Comput..

[43] Ho SY, Chen JH, Huang MH (2004). Inheritable genetic algorithm for biobjective 0/1 combinatorial optimization problems and its applications. IEEE Trans. Syst. Man Cybern. Part B Cybern..

[44] Ho, S.-Y. in *Proc. Genetic and Evolutionary Computation Conference, Orlando, Florida, USA, 1999.*

[45] Wang J-R (2017). ESA-UbiSite: accurate prediction of human ubiquitination sites by identifying a set of effective negatives. Bioinformatics.

[46] Tsai M-J (2020). GREMA: modelling of emulated gene regulatory networks with confidence levels based on evolutionary intelligence to cope with the underdetermined problem. Bioinformatics.

[47] Ho S-Y, Huang H-L (2001). Facial modeling from an uncalibrated face image using a coarse-to-fine genetic algorithm. Pattern Recogn..

[48] Yerukala Sathipati S, Ho S-Y (2018). Identifying a miRNA signature for predicting the stage of breast cancer. Sci. Rep..

[49] Zhou, D., Fang, J., Song, X., Guan, C. & Yang, R. J. I. IoU Loss for 2D/3D Object Detection. (2019).

[50] Gevorgyan, Z. SIoU Loss: More Powerful Learning for Bounding Box Regression. Preprint https://arxiv.org/abs/2205.12740 (2022).

[51] Du S, Zhang B, Zhang P (2021). Scale-sensitive IOU loss: An improved regression loss function in remote sensing object detection. IEEE Access.

